# Genetic footprints of assortative mating in the Japanese population

**DOI:** 10.1038/s41562-022-01438-z

**Published:** 2022-09-22

**Authors:** Kenichi Yamamoto, Kyuto Sonehara, Shinichi Namba, Takahiro Konuma, Hironori Masuko, Satoru Miyawaki, The BioBank Japan Project, Yoichiro Kamatani, Nobuyuki Hizawa, Keiichi Ozono, Loic Yengo, Yukinori Okada

**Affiliations:** 1 Department of Statistical Genetics, Osaka University Graduate School of Medicine, Suita, Japan; 2 Department of Pediatrics, Osaka University Graduate School of Medicine, Suita, Japan; 3 Laboratory of Statistical Immunology, Immunology Frontier Research Center (WPI-IFReC), Osaka University, Suita, Japan; 4 Integrated Frontier Research for Medical Science Division, Institute for Open and Transdisciplinary Research Initiatives, Osaka University, Suita, Japan; 5 Department of Pulmonary Medicine, Faculty of Medicine, University of Tsukuba, Tsukuba, Japan; 6 Department of Neurosurgery, Faculty of Medicine, The University of Tokyo, Tokyo, Japan; 7 Laboratory of Complex Trait Genomics, Department of Computational Biology and Medical Sciences, Graduate School of Frontier Sciences, The University of Tokyo, Tokyo, Japan; 8 Institute for Molecular Bioscience, The University of Queensland, Brisbane, Queensland Australia; 9 Laboratory for Systems Genetics, RIKEN Center for Integrative Medical Sciences, Yokohama, Japan; 10 Center for Infectious Disease Education and Research, Osaka University (CiDER), Suita, Japan; 11 Department of Genome Informatics, Graduate School of Medicine, The University of Tokyo, Tokyo, Japan

**Keywords:** Genetic variation, Social evolution, Genome-wide association studies

## Abstract

Assortative mating (AM) is a pattern characterized by phenotypic similarities between mating partners. Detecting the evidence of AM has been challenging due to the lack of large-scale datasets that include phenotypic data on both partners, especially in populations of non-European ancestries. Gametic phase disequilibrium between trait-associated alleles is a signature of parental AM on a polygenic trait, which can be detected even without partner data. Here, using polygenic scores for 81 traits in the Japanese population using BioBank Japan Project genome-wide association studies data (*n* = 172,270), we found evidence of AM on the liability to type 2 diabetes and coronary artery disease, as well as on dietary habits. In cross-population comparison using United Kingdom Biobank data (*n* = 337,139) we found shared but heterogeneous impacts of AM between populations.

## Abstract (web summary)

Yamamoto et al. find genetic evidence of assortative mating based on dietary habits and disease phenotypes in the Japanese population, and show that this pattern of partner choice is markedly different from its European-ancestry counterpart.

## Main

Positive assortative mating (AM) is a commonly observed phenomenon in human mating, where individuals with similar phenotypes are more likely to form partnerships than expected by chance (that is, random mating)^1^. Partner similarities involve a wide range of factors: age, geographical factors, racial/ethnic background, religion, socioeconomic status and educational background, as well as physical, personality and psychological traits^1,2^, and AM for many of these traits has been demonstrated by comparing partner phenotypes^3–8^. In the field of population genetics, we know that AM increases the homozygosity of genotypes of the trait-associated variants, induces long-range correlations between alleles across the genome and increases genetically determined variance of the traits in a population scale^9^.

Quantitative impacts of AM in human genetics have been investigated by focusing on the deviation from the Hardy–Weinberg equilibrium (HWE) in trait-associated variants. However, this approach requires large sample sizes, especially when the effect sizes of the associated variants are small. Furthermore, ancestral endogamy (mating within the limits of a specific social group) could confound these relationships^10–12^. An alternative approach is to study genetic similarities between partners. This approach revealed the existence of AM in Europeans on anthropometric traits (height and body mass index (BMI)), and social and behavioural phenotypes (educational attainment and alcohol consumption)^13–17^. Although partner genotype–phenotype data have been analysed in these studies, it has been relatively challenging to achieve biobank-scale sample sizes in populations of diverse ancestries.

Recently, Yengo et al. developed a new method to quantify the impact of AM using data from large-scale genome-wide association studies (GWAS) without partner data^18^. The authors focused on the gametic phase disequilibrium (GPD) between trait-associated alleles^19^. Under AM, physically distant trait-associated alleles correlate with each other beyond local linkage disequilibrium (LD) in polygenic traits. Thus, the genetic effects of AM from parents are reflected as the correlation between two polygenic scores (PGS) from physically distant sets of chromosomes (for example, PGS from odd-numbered chromosomes, PGS_odd_, and that from even-numbered chromosomes, PGS_even_). The application of PGS to United Kingdom Biobank (UKB) GWAS data has provided evidence of AM for adult height and educational background, and the researchers further validated these results via AM estimation using spousal pairs. While this method has advantages in that it only requires GWAS data without partner information, its applications have so far been limited to European-ancestry populations. More generally, there have been very few investigations of the genetic effects of AM outside of the European-ancestry populations.

Here, we report a PGS-based analysis of AM in a Japanese cohort using BioBank Japan Project (BBJ) GWAS data, one of the largest non-European biobanks with deep phenotype information^20^. We estimate the AM-induced GPD across 81 human complex traits by calculating the correlation between PGS_odd_ and PGS_even_ with robust adjustments for population stratification. We then compare our results with those derived from the UKB genotype–phenotype data. Our study provides evidence of AM in the previous generation of the current Japanese cohort, and highlights the importance of studying AM in populations representative of non-European ancestries.

## Results

### Study overview

**Fig. 1:**
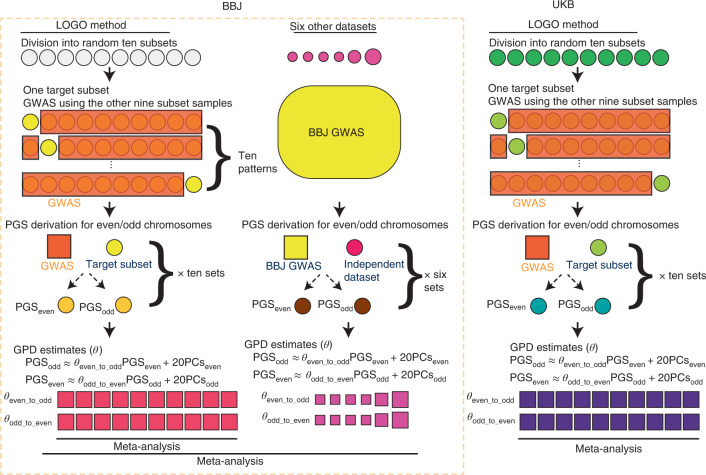
An overview of the study design. We randomly divided the BBJ mainland samples into ten subsets to apply the LOGO method. We conducted GWAS using training samples and withholding the target subset using GCTA-fastGWA. We derived PGS for even-/odd-numbered chromosomes (PGS_odd_/PGS_even_) in the target subset using the PRS-CS method and estimated GPD for even-/odd-numbered chromosomes (*θ*_even_to_odd_ and *θ*_odd_to_even_). We then meta-analysed the GPD estimates across the ten subsets. For the six independent Japanese or EAS cohorts, we derived PGS_odd_/PGS_even_ based on fastGWA results from the whole mainland sample in BBJ. Finally, we performed a meta-analysis of the GPD estimates across all EAS datasets (*n* = 172,270). We adopted the same LOGO method to estimate GPD in the UKB data (*n* = 337,139).

As biobank-scale GWAS results for multiple traits in East Asian ancestry populations (EAS) are not publicly available, we adopted the tenfold leave-one-group-out (LOGO) meta-analysis method for BBJ to estimate AM-induced GPD^21^ (see Fig. 1 for an overview). In brief, we selected individuals from the BBJ mainland cluster (*n* = 156,151) for phenotypic uniformity (Supplementary Table 1 and Supplementary Fig. 1)^22^ and then randomly separated individuals into ten subsets. For each of the target subsets, we conducted GWAS in the other nine subgroups using GCTA-fastGWA, a mixed linear model (MLM) approach to control for population stratification and relatedness^23,24^. Then, we calculated PGS_odd_ and PGS_even_ for the individuals in the target subset using the posterior variant effect sizes inferred by PRS-CS^25^ from GWAS results generated with GCTA-fastGWA. We estimated GPD for each trait from correlations between PGS_odd_ and PGS_even_ (*θ*_even_to_odd_ and *θ*_odd_to_even_) adjusted for 20 principal components (PCs) derived from odd-/even-numbered chromosomes (PCs_odd/even_) to correct for population stratification (see details in Methods). Finally, we meta-analysed the GPD estimates across ten subsets.

We replicated our findings in six independent cohorts of East Asian ancestry: *n* = 8,947 from the BBJ Ryukyu cluster, *n* = 1,275 from the Osaka University healthy cohort, two datasets from the Nagahama cohort study (*n* = 1,543 as Nagahama_1 and *n* = 1,452 as Nagahama_2), *n* = 1,110 from the Japan Biological Informatics Consortium (JBIC) and *n* = 1,842 from UKB EAS. For each cohort, we derived PGS_odd_ and PGS_even_ using the whole-sample MLM-GWAS in the BBJ mainland cluster, and estimated the GPD from correlations between PGS_even_ and PGS_odd_ as described above. Finally, we meta-analysed the GPD estimates across all EAS. Regarding the UKB data, we applied the LOGO method and quantified GPD in the same way as described in the BBJ part (*n* = 337,139).

### GPD analysis across 81 complex traits in the Japanese population

We estimated GPD across 81 complex traits measured in BBJ participants (57 anthropometric and biomarker traits, 17 dietary habits and behavioural traits, six diseases and one negative control; Supplementary Tables 2–5). As *θ*_even_to_odd_ and *θ*_odd_to_even_ values of each trait were similar but not completely identical due to the difference in the single nucleotide polymorphism (SNP) selected for PGS calculation, we conservatively adopted the value with larger variance as the GPD estimate of the trait (*θ*). We set a study-wide significance threshold at *P* = 6.2 × 10^−4^ (= 0.05/81) by applying Bonferroni’s correction based on the number of traits analysed.

**Fig. 2:**
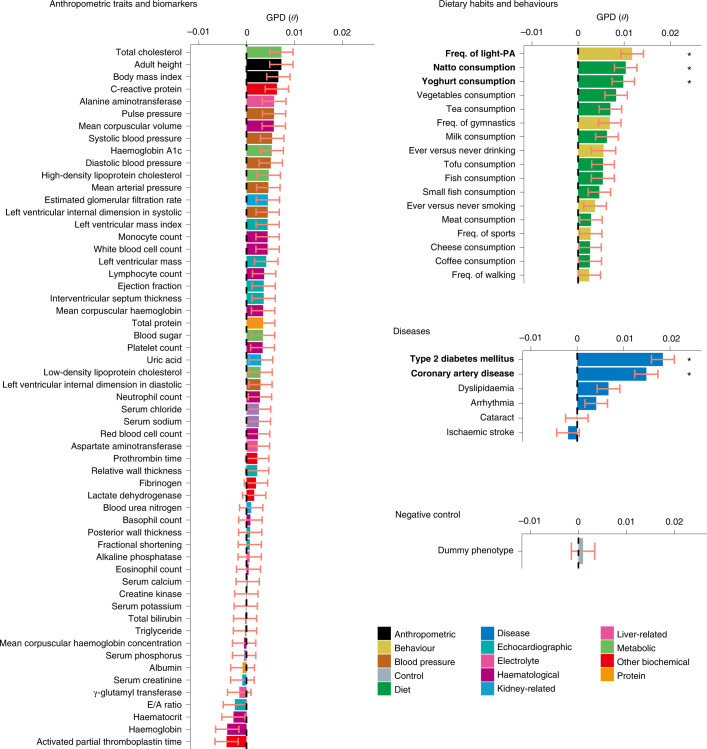
Estimates of GPD for 81 complex traits in the Japanese population. For 81 human complex traits, we quantified GPD as the correlation between trait-specific PGSs for odd-/even-numbered chromosomes. We selected the meta-analysed GPD estimate (*θ*) with the larger variance between *θ*_even_to_odd_ and *θ*_odd_to_even_ in all Japanese or EAS cohorts (*n* = 172,270). *P* values were determined by two-sided Wald test. We set a study-wide significance threshold at *P* < 6.2 × 10^−4^ (=0.05/81) by applying Bonferroni’s correction for multiple comparison. Statistically significant traits are marked with an asterisk and in bold. Detailed results are presented in Supplementary Table 6. The bar plots represent the point estimates, and error bars represent the s.e. Freq., frequency.

We detected significant GPD estimates in five traits. The most significant trait was type 2 diabetes (T2D; *θ*_T2D_ = 0.018, standard error (s.e.) = 0.0025, *P* = 5.2 × 10^−14^; Fig. 2, Table 1 and Supplementary Table 6), followed by coronary artery disease (CAD; *θ*_CAD_ = 0.015, s.e. = 0.0025, *P* = 2.2 × 10^−9^). Among dietary and behavioural traits, we detected significant GPD estimates for the frequency of light physical activity (light-PA; *θ*_light-PA_ = 0.012, s.e. = 0.0025, *P* = 2.0 × 10^−6^), natto (*θ*_natto_ = 0.010, s.e. = 0.0024, *P* = 2.4 × 10^−5^) and yoghurt consumption (*θ*_yoghurt_ = 0.010, s.e. = 0.0024, *P* = 5.6 × 10^−5^). We did not detect significant evidence of AM on alcohol consumption and smoking status (*θ*_alcohol_ = 0.006, s.e. = 0.0026, *P* = 0.04 and *θ*_smoking_ = 0.004, s.e. = 0.0025, *P* = 0.14), which have been previously reported in other studies^14,16^.

**Table 1:** A list of traits with a significant GPD estimate in the Japanese and EAS meta-analysis

Trait	Category	*θ*	s.e.	*P* value
Type 2 diabetes	Disease	0.018	0.0025	5.2 × 10^−14^
Coronary artery disease	Disease	0.015	0.0025	2.2 × 10^−9^
Frequency of light physical activity	Behaviour	0.012	0.0025	2.0 × 10^−6^
Natto consumption	Diet	0.010	0.0024	2.4 × 10^−5^
Yoghurt consumption	Diet	0.010	0.0024	5.6 × 10^−5^

Full results for all traits are listed in Supplementary Table 6.

*P* values were determined by two-sided Wald test. We set a study-wide significance threshold at *P* < 6.2 × 10^−4^ (=0.05/81) by applying Bonferroni’s correction for multiple comparison.

In summary, in our biobank-based analyses we found robust genetic evidence of AM in the Japanese population, mostly observed in cardiometabolic diseases and dietary habits.

### Transchromosomal characteristics of GPD estimates

**Fig. 3:**
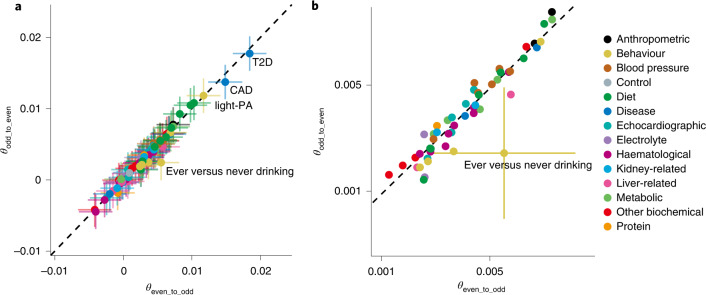
Correlations between GPD estimates from even to odd chromosomes and GPD estimates from odd to even chromosomes for 81 traits in BBJ. **a**, The correlation plot of 81 traits between *θ*_even_to_odd_ and *θ*_odd_to_even_ in the Japanese population. **b**, The enlarged plot of **a** around the trait of ever versus never drinking. The *x* axis indicates the meta-analysed GPD estimated from even to odd chromosomes (*θ*_even_to_odd_), and the *y* axis indicates that from odd to even chromosomes (*θ*_odd_to_even_). The error bars represent s.e. The dashed line represents *θ*_odd_to_even_ = *θ*_even_to_odd_.

Next, we compared *θ*_odd_to_even_ and *θ*_even_to_odd_, the GPD estimates from regression of PGS_odd_ onto PGS_even_, and that of PGS_even_ onto PGS_odd_, under robust controls for population stratification (Fig. 3). Although *θ*_odd_to_even_ and *θ*_even_to_odd_ were similar for almost all traits, we detected a notable difference for the history of alcohol consumption (*θ*_odd_to_even_ = 0.002 but *θ*_even_to_odd_ = 0.006 in ever versus never drinking, Grubbs test *P* = 4.6 × 10^−7^). This observation can be explained by the genetic architecture of alcohol-related behaviours, which involves a subset of variants with strong effects in EAS populations. These variants are mainly located on even-numbered chromosomes (that is, *GCKR* on chromosome 2, *ADH1B* on chromosome 4 and *ALDH2* on chromosome 12)^26^, and they are related to natural selection and population stratification in EAS and the Japanese^27,28^. Thus, there is a stronger correlation between PGS_even_ and PCs_even_ than between PGS_odd_ and PCs_odd_ (Supplementary Fig. 3): namely, even-chromosome-specific correlation of alcohol consumption PGS and population stratification. Collinearity in the multivariate regression model destabilized the even to odd GPD estimate, resulting in transchromosomal imbalance. We did not detect strong LD nor excess homozygosity at these top four variants associated with alcohol consumption (*R*^2^ < 0.1 for each variant pair, and inbreeding coefficient (*F*) < 0.1 at each variant)^26^.

### Cross-population comparison of AM using UKB data

**Fig. 4:**
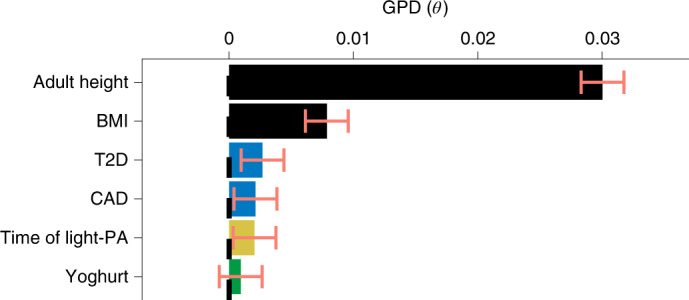
GPD estimates for six complex traits in the UKB. For six complex traits, we quantified GPD as the correlation between trait-specific PGSs for odd-/even-numbered chromosomes. GPD estimate (*θ*) with the larger variance between *θ*_even_to_odd_ and *θ*_odd_to_even_ was selected in white British individuals from UKB data (*n* = 337,139). The bar plots represent the point estimates, and error bars represent the s.e.

We estimated GPD for six traits in the UKB GWAS data: T2D, CAD, light-PA and yoghurt consumption as traits with significant AM signatures in the Japanese, and adult height and obesity (BMI) as gold standard controls for AM (Fig. 4). We robustly replicated the GPD estimate for adult height in the European-ancestry population as a sanity check (*θ*_height in UKB_ = 0.030 and 0.030 for the current and previous studies^18^, respectively). The GPD estimate of BMI in our work was slightly higher than in the previous study (*θ*_BMI in UKB_ = 0.0079 and 0.0001 for the current and previous studies^18^, respectively). It is noteworthy that the GPD estimates for adult height were relatively higher than those for BMI in both European-ancestry and Japanese populations (that is, *θ*_height in EAS_ = 0.0073 and *θ*_BMI in EAS_ = 0.0067). However, the GPD estimate for adult height was not as high in Japanese compared with the European-ancestry cohort. This result was consistent with previous epidemiological reports^5^, in which the correlation of height between spousal pairs in Western countries was higher than those in non-Western regions. We note that height was one of the traits with the strongest positive natural selection among Europeans^29^, whereas it was not in the Japanese^27,28^. The GPD estimates of T2D, CAD, light-PA and yoghurt consumption were higher in Japanese than in European-ancestry populations (*θ*_T2D in EAS_ = 0.018 versus *θ*_*T2D in UKB*_ = 0.003, *θ*_CAD in EAS_ = 0.014 versus *θ*_CAD in UKB_ = 0.002, *θ*_light-PA in EAS_ = 0.012 versus *θ*_light-PA in UKB_ = 0.002, *θ*_*y*__oghurt in EAS_ = 0.010 versus *θ*_*y*__oghurt in UKB_ = 0.001). This result suggests a population-specific effect of AM.

### Sensitivity analyses

We performed sensitivity analyses to confirm the robustness of our findings. We investigated the potential effect of cryptic population stratification of the Japanese population in two ways. First, we simulated a heritable dummy phenotype as a negative control (see details in Methods). Regarding the GPD estimates of the dummy phenotype, we could not observe transchromosomal correlation (*θ*_dummy_ = 0.0010, s.e. = 0.0024, *P* = 0.69; Fig. 1). Second, we sequentially changed the number of the PCs used for the adjustment of PGSs from 0 to 30 for the traits with significant AM (T2D, CAD, light-PA, natto consumption and yoghurt consumption). We confirmed that the GPD estimates did not apparently change when varying the number of PCs (Supplementary Fig. 4).

**Fig. 5:**
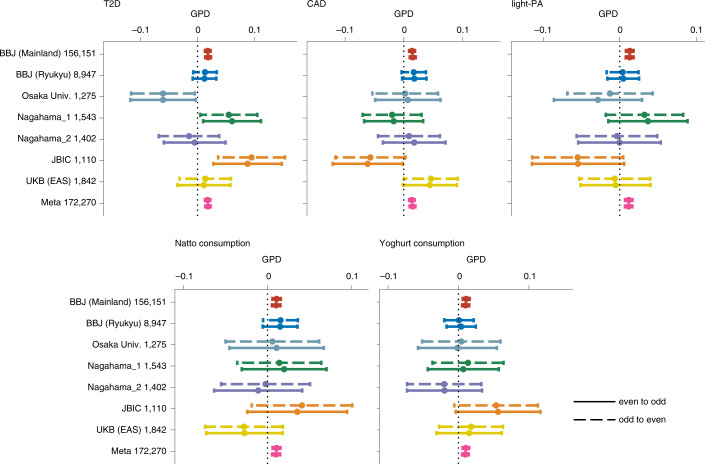
Forest plots of GPD estimates of five traits related to AM. Forest plots of five significant traits, T2D, CAD, light-PA, natto consumption and yoghurt consumption. For all plots, a given label and number along vertical axes represent the name and the sample size of the cohort, respectively. The points indicate the point estimates, and error bars indicate 95% confidence intervals. Osaka Univ., Osaka University healthy cohort; Meta, meta-analysis.

The positive GPD estimates in the significant traits were not always consistent between cohorts (Fig. 5 and *P*_Het_ in Supplementary Table 6). We varied the grouping of the chromosomes in different ways as (1) first half and second half, and (2) pseudo-random (see details in Methods). We estimated the meta-analysed GPD for significant traits and confirmed that there was no apparent difference in the GPD estimates between the original grouping and alternative groupings (Supplementary Fig. 5).

Next, we compared our observed GPD estimate with the theoretical expectation described in the original study (see details in Methods)^18^. The expected value of GPD depends on various parameters: phenotypic correlation between partners (*r*), equilibrium heritability ($h_{\mathrm{{eq}}}^2$), SNP-based heritability ($h_{\mathrm{{snp}}}^2$), the number of causal variants (*M*) and sample size (*n*). Based on *r* and $h_{\mathrm{{eq}}}^2$ estimated from published studies^30–35^, $h_{\mathrm{{snp}}}^2$ estimated from GREML-LDMS, *M* assumed between 10,000 and 100,000 and *n* from the reference GWAS size, we calculated the expected GPD in the dummy data, adult height, BMI, T2D and CAD (Supplementary Table 7). Although part of the tested data showed a match between the theoretical and the observed GPD values, there were mismatches, which we believe are due to the parameter dependence of the theoretical GPD.

Finally, we considered the impact of geographical factors not captured by PCs for partner similarities and AM^36^. First, we assessed regional differences in the 81 complex traits based on the registered area information in the BBJ mainland (from northeast to southwest) and detected strong regional differences in light-PA, natto consumption, yoghurt consumption, T2D and CAD. To correct for the influence of the regional differences in GPD estimates, we adopted the leave-one-region-out (LORO) approach (see details in Supplementary Note). After the LORO approach, the GPD estimates of T2D, CAD and vegetable consumption were statistically significant (Supplementary Fig. 7). These significant GPD estimates in T2D, CAD and vegetable consumption might reflect the effects of parental AM not influenced by the geographical factors.

## Discussion

In this study, we investigated genetic footprints of AM for 81 complex traits in the Japanese population using a PGS-based approach^18^. Our study successfully detected significant GPD among alleles associated with five human complex traits, with T2D showing the strongest AM signature. Our cross-population comparisons using the UKB data suggest shared AM signatures between Japanese and European-ancestry populations, but with heterogeneous impacts among traits. We further found that accounting for geographical factors could improve the robustness of the results in the Japanese sample.

Previous studies have reported spousal concordance of T2D and CAD in populations of East Asian ancestries^34,37,38^. Our results suggest that mate choice (as opposed to shared environmental factors such as urbanization or local culture) on traits associated with the liability to these diseases could be the cause of the observational similarity between partners.

Several behavioural and dietary habits showed significant AM signatures: light-PA, natto, yoghurt and vegetable consumption. Natto consumption is a unique dietary habit of East Asian and Japanese populations. Our results suggest behavioural and dietary habits are a driving force of AM in Japan, and dietary habits are known to be involved in the natural selection pressure of Japanese^27,28^. Population admixture and natural selection represent other potential causes of GPD, and they are entangled with AM. Although admixture and population stratification could create positive GPD, we empirically assessed the potential cause by robustly controlling for sample selection and population stratification. As natural stabilizing selection would induce a negative correlation between alleles and lead to negative GPD, it would result in an opposite effect to AM^39^.

Applying the LORO approach as an adjustment for geography-related effects not captured by PCs, we detected statistically significant GPD estimates in T2D, CAD and vegetable consumption. Given the difference in the geographical distribution in many traits and the decrease of the GPD estimates when using the LORO approach, our initial GPD estimates could reflect not only AM, but also include the effects of social homogamy related to geography. This genetic correlation induced by geography-related effect could be especially high in natto and yoghurt consumption. This insight corroborates the results provided by Okbay et al.^40^, in which partner PGS correlation would be influenced by background geographical factors not captured by PCs.

Our study has several limitations. One potential limitation is that we did not assess AM in educational attainment (EA), as such information was not collected in BBJ, a hospital-based biobank. In European-ancestry populations, EA is among the traits strongly influencing AM^13,18^, but it is also associated with common diseases and cognitive traits^40^. Observational studies in the Japanese population have likewise reported both educational AM and associations between EA and cardiometabolic diseases and dietary habits^41–43^. On the other hand, other studies found that spousal similarities in cardiometabolic diseases were independent of EA^34,38^. As our results could reflect the potential involvement of EA, further work will be required in Japanese cohorts, including data on educational attainment.

Furthermore, we could not conduct a confirmation analysis using spousal or partner data due to the lack of available biobank-scale data. We therefore have not examined correlations between our GPD estimates and partner genetic similarities. As BBJ is a hospital-based medical cohort, the distribution of phenotypes and genotypes may not fully reflect that of a healthy population. Furthermore, due to the lack of information on birth places or residences in BBJ, our results may not fully account for geographical differences. Large sample size differences between geographical regions (*n* = 7,645 in Hokkaido to *n* = 91,743 in Kanto-Koshinetsu) could also affect the power of GWAS and the estimation of local GPD using our LORO approach. While some social homogamy (such as geographical proximity) and AM could not be completely independent^40,44,45^, these limitations could be mitigated through future enhancement of cohorts and collaborations.

In summary, we found genetic evidence of AM in the Japanese population for a set of complex traits, using the PGSs-based approach and large-scale biobank data. Our results contribute to our understanding of AM in humans and warrant further investigations of AM in populations of more diverse ancestries.

## Methods

### Study cohort description

We used data on a total of 172,270 individuals of Japanese and East Asian ancestry. Of these, data on 165,098 individuals were obtained from BBJ, which has enrolled ≥200,000 participants to date. BBJ is a multi-institutional hospital-based genome cohort that collected participants affected with at least one of 47 diseases^20^. We excluded (1) individuals with low genotyping call rates (<98%), (2) closely related individuals (PI_HAT ≥ 0.125 by PLINK, v.1.90b4.4; https://www.cog-genomics.org/plink/) and (3) outliers from the Japanese cluster based on principal component analysis (PCA) using PLINK2 (v.2.00a2.3 and v.2.00a3; https://www.cog-genomics.org/plink/2.0/) with samples of the 1000 Genomes Projects. Further, we separated the BBJ individuals into two Japanese clusters^22,27^ the mainland cluster (*n* = 156,151) and Ryukyu cluster (*n* = 8,947), by visual inspection based on the PCA plot (Supplementary Fig. 1). All the participants provided written informed consent approved from ethics committees of RIKEN Center for Integrative Medical Sciences, and the Institute of Medical Sciences, the University of Tokyo.

The Japanese subjects in replication cohorts were collected from three Japanese population-based cohorts (the Nagahama cohort study, JBIC and the Osaka University healthy cohort). The Nagahama cohort study is a community-based cohort in Nagahama city, Shiga prefecture, Japan. The study recruited healthy individuals between the ages of 30 and 74 (ref. ^46^). JBIC consists of Epstein–Barr virus-transformed B lymphoblast cell lines of unrelated Japanese individuals^47^. Osaka University healthy cohort is a volunteer-based cohort study recruited from the Osaka University Graduate School of Medicine, the University of Tokyo and the University of Tsukuba^48^. For each cohort, we also excluded individuals with a low genotyping call rate, a high heterozygosity rate, closely related individuals (PI_HAT ≥ 0.125) and PCA outliers from EAS populations^28,48,49^. In addition, we extracted the EAS individuals from UKB. UKB is a population-based cohort that recruited approximately 500,000 individuals between 40 and 69 years of age from across the United Kingdom^50^. We obtained EAS individuals from unrelated UKB individuals based on PCA visualization combined with the 1000 Genomes Projects (Supplementary Fig. 2). Finally, we included 16,119 individuals in the replication study (*n* = 8,947 from BBJ Ryukyu, *n* = 1,275 from Osaka University healthy cohort, *n* = 2,945 from the Nagahama cohort study, *n* = 1,110 from JBIC and *n* = 1,842 from UKB EAS). This study was approved by the ethical committee of Osaka University Graduate School of Medicine.

### Phenotype curation

BBJ collected baseline clinical information and dietary and activity habits information through interviews and reviews of medical records using a standardized questionnaire. We selected 81 traits (57 anthropometric traits and biomarkers, 11 dietary habits, six behavioural traits, six diseases and one dummy; Supplementary Tables 2–4). We used these data from participants above the age of 18, and drinking and smoking traits from those above the age of 20. We normalized each anthropometric trait and biomarker traits by applying rank-based inverse normal transformation as previously reported (Supplementary Table 8)^51–53^. For each dietary habit, the participants were asked to clarify the frequency of consumption on a four-point scale, and we assigned the corresponding values to their responses as previously described^26^, where almost every day = 7, 3–4 days per week = 3.5, 1–2 days per week = 1.5 and rarely = 0. Behavioural traits included ever versus never drinking and ever versus never smoking^54^ as binary traits, and the frequency of four PAs (light-PA, gymnastics, walking and sports). For each PA, participants were also asked for the frequency and the length of time per week on a seven-point scale, and we quantified the activity by converting the responses to total minutes of activity time per week (min week^–1^), where ≥30 (15) min day^–1^ = 210 (105), <30 (15) min day^–1^ = 140 (70), three to four times a week for ≥30 (15) min = 105 (52.5), three to four times a week for <30 (15) min = 70 (35), one to two times a week for ≥30 (15) min = 45 (22.5), one to two times a week for <30 (15) min = 30 (15) and rarely = 0 (the number in parentheses indicates gymnastics time).

For disease phenotypes, cases with myocardial infarction, stable angina and unstable angina were reclassified as CAD. We then selected six diseases from the target disease of BBJ (T2D, dyslipidaemia, cataract, CAD, arrhythmia and ischaemic stroke), where the number of cases exceeded 10,000 individuals^55^.

In addition, we set a dummy phenotype as a negative control. We generated a phenotype with heritability (*h*^2^ = 0.5) from 10,000 causal variants randomly sampled from BBJ GWAS data using GCTA GWAS simulation^56^. The phenotype followed the model *y*_*j*_ = *g*_*j*_ + *e*_*j*_, where *g*_*j*_ = Σ_*i*_(*W*_*ij*_*β*_*i*_) and *W*_*ij*_ = (*x*_*ij*_ – 2*p*_*i*_)[2*p*_*i*_(1 – *p*_*i*_)]^−1/2^, where *x*_*ij*_ is the genotype for the *i*th causal variant of the *j*th individual, *p*_*i*_ is the allele frequency of the *i*th causal variant within a population and *e*_*j*_ is the residual effect generated from a normal distribution with mean 0 and variance Var(*g*_*j*_)(1 − *h*^2^)/*h*^2^. *β*_*i*_ is the effect size of the *i*th causal variant generated from a normal distribution with mean 0 and variance 1 (ref. ^57^). The values were normalized by applying a rank-based inverse normal transformation.

### Genotyping, quality control and imputation of GWAS data

The BBJ GWAS data were genotyped using the Illumina HumanOmniExpressExome BeadChip or a combination of the Illumina HumanOmniExpress and HumanExome BeadChips. The quality control of the genotypes was described elsewhere^51^. In brief, we excluded variants satisfying the following criteria: (1) call rate <99%, (2) *P* value for HWE < 1.0 × 10^−6^, (3) number of heterozygotes <5 and (4) a concordance rate <99.5% or a non-reference concordance rate between the GWAS array and whole genome sequencing. The genotype data were phased by Eagle (v.2; https://alkesgroup.broadinstitute.org/Eagle/), and imputed with the 1000 Genomes Project Phase3 (v.5) and BBJ1K using Minimac3 software (v.2.0.1; https://genome.sph.umich.edu/wiki/Minimac3). After imputation, we excluded variants with an imputation quality of R-square (Rsq) < 0.7 and those with a minor allele frequency (MAF) < 1%.

As for the other Japanese datasets, JBIC was genotyped using Illumina HumanCoreExome Beadchip. As stringent quality control filters, we excluded the variants that satisfied (1) call rate < 0.99, (2) MAF < 1% and (3) HWE *P* < 1.0 × 10^−7^ (ref. ^47^). Osaka University healthy cohort was genotyped using Illumina Infinium Asian Screening Array. We excluded the variants that satisfied (1) call rate < 0.99, (2) minor allele count < 5 and (3) HWE *P* < 1.0 × 10^−5^ (ref. ^48^). The Nagahama cohort study was genotyped using six genotype arrays. We then selected two platforms (Illumina Human610-Quad Beadchip and Illumina HumanOmni2.5-4v1 Beadchip) with a large number of samples. For each of the two datasets, we excluded variants with (1) call rate < 0.98, (2) MAF < 1% and (3) HWE *P* < 1.0 × 10^−6^ (ref. ^28^). Genotype data were phased by Shapeit (v.2; https://mathgen.stats.ox.ac.uk/genetics_software/shapeit/shapeit.html) or Eagle, and imputed with the reference panel from the 1000 Genomes Project Phase3 (v.5) and BBJ1K using Mimimac3. After imputation, we excluded variants with an imputation quality of Rsq < 0.7 and MAF < 1%.

The UKB project was genotyped using either Applied Biosystems UK BiLEVE Axiom Array or Applied Biosystems UKB Axiom Array. The genotypes were imputed using the Haplotype Reference Consortium, UK10K and the 1000 Genomes Phase 3 reference panel by IMPUTE4. The detailed characteristics of the cohort and genotype–phenotype data were described elsewhere^50^. We extracted EAS individuals and excluded variants with INFO score ≤0.8 and MAF ≤ 1%.

### GWAS

As independent external reference GWASs or genotype data of Japanese ancestry were not publicly available, we adopted a tenfold LOGO meta-analysis to maintain both the accuracy of the GWAS statistics and the statistical power in PGS^21^. We first randomly split the BBJ mainland samples into the 10 target subsets. GWAS was performed on 81 complex traits for samples excluding the target subset using GCTA-fastGWA (v.1.93.3beta2; https://cnsgenomics.com/software/gcta/#Overview) as a MLM approach with 7,401,847 autosomal variants^23,24^. For GCTA-fastGWA, we computed a sparse genetic relationship matrix (GRM) for BBJ participants (*n* = 182,961) using slightly LD-pruning variants (LD-pruning parameters in PLINK: –indep-pairwise 1000 100 0.9, and MAF ≥ 1%, sparse GRM parameter: –make-bK-sparse 0.05). Regarding the 57 anthropometric traits and biomarkers, the 11 dietary traits, the four PA traits and the two binary traits in the behavioural traits, we fitted age, age-squared, sex, the top 20 PCs and 47 disease status as covariates. For the six diseases, we also fitted age, age-squared, sex and the top 20 PCs as covariates. We also performed GWAS using GCTA-fastGWA for all individuals in the BBJ mainland cluster to apply to other Japanese or EAS datasets. LD score regression (LDSC, v.1.0.0; https://github.com/bulik/ldsc) was applied to the summary statistics of the whole-sample GWAS to estimate potential population stratification. We adopted the HapMap3 SNPs, excluding the human leukocyte antigen region, using precomputed LD scores from 1KG EAS downloaded from the LDSC software website (Supplementary Table 5)^58^.

To estimate phenotypic variances explained by imputed data for some of the traits, we applied GREML-LDMS using GCTA (v.1.93.2beta; https://cnsgenomics.com/software/gcta/#Overview)^57^. We created the GRM using all variants for BBJ mainland samples. We estimated LD scores using default parameters in GCTA, and stratified SNPs into LD score quartiles. Next, we divided the SNPs within each LD score quartile into six MAF groups (MAF < 5%, 5% ≤ MAF < 10%, 10% ≤ MAF < 20%, 20% ≤ MAF < 30%, 30% ≤ MAF < 40%, 40% ≤ MAF) and generated 24 GRMs. We calculated the phenotypic variance for each GRM and summed them to derive the total phenotypic variance (Supplementary Table 7). In the calculations, we randomly sampled 50,000 unrelated individuals (GRM < 0.05) randomly downsampled from BBJ mainland individuals to avoid computational burden and used the same normalized values for quantitative traits and covariates for binary traits as used in the GWAS analysis.

### Polygenic risk score derivation and GPD estimation

To derive PGSs of individuals in each of the target subsets, we applied PRS-CS (https://github.com/getian107/PRScs) to construct PGSs that included genome-wide HapMap3 variants. PRS-CS is one of the beta shrinkage methods, which applies a Bayesian regression framework to identify posterior variant effect sizes based on continuous shrinkage before using both GWAS summary data and the external LD reference panel^25^. When the training sample size was large enough and the case–control imbalance was small, the automated optimization model (PRS-CS-auto) had the same precision as the grid model^59,60^. Therefore, for each of the target folds, we estimated the posterior mean effects of SNPs from the MLM-GWAS summary data of all training samples using PRS-CS-auto with the precomputed HapMap3 SNP LD reference panel from 1KG EAS downloaded from the PRS-CS website. We calculated PGS_odd_ and PGS_even_ of individuals within the target subset using the estimated posterior effect of SNPs by PLINK2 score function. We normalized the calculated PGSs for each trait in each target subset to compare the effect sizes across the phenotypes.

We quantified the trait variance explained by the derived PGSs in individuals within one withheld subgroup. Each trait was modelled as a combination of PGS and all covariates. The null hypothesis used the same model without the PGS term. We calculated the adjusted *R*^2^ for quantitative traits and the Nagelkerke’s *R*^2^ for binary traits (Supplementary Table 5).

For GPD estimation, we performed PCA of even and odd number chromosomes for each of the target subsets. We then estimated GPD using a linear regression method following the formula based on the original study^18^:

$${\mathrm{PGS}_{\rm{odd}}}\approx \theta _{\mathrm{{even}}\_{\mathrm{to}}\_{\mathrm{odd}}}{\mathrm{PGS}_{\rm{even}}} + 20{\mathrm{PCs}_{\rm{even}}}$$

$${\mathrm{PGS}_{\rm{even}}}\approx \theta _{\mathrm{{odd}}\_{\mathrm{to}}\_{\mathrm{even}}}{\mathrm{PGS}_{\rm{odd}}} + 20{\mathrm{PCs}_{\rm{odd}}}$$

where PGS is the scaled polygenic score, PCs are the results of the PCA and *θ* is the estimate of GPD. We further meta-analysed the GPD estimate from each of the ten subsets using the fixed effect method using metafor (v.1.9-9; http://www.metafor-project.org/doku.php/metafor) implemented in *R* (v.3.4.0; https://www.r-project.org/). We also estimated the GPD for the other Japanese and EAS datasets using the summary results of the whole BBJ sample GWASs by PRS-CS-auto. Finally, we performed a meta-analysis on the GPD estimates from the BBJ and other Japanese and EAS datasets by the fixed effect method using metafor. We estimated the *P* value of meta-analysed GPD using the Wald test.

To assess the robustness of our analysis to the chosen grouping of chromosomes, we altered the combinations of chromosomes such that the number of SNPs was the same in the two groups: (1) first half and second half; chromosomes 1–8 versus chromosomes 9–22, and (2) pseudo-random chromosomes; chromosomes 1, 3, 5, 6, 9, 10, 13, 14, 17 and 18 versus chromosomes 2, 4, 7, 8, 11, 12, 15, 16, 19, 20, 21 and 22. Using the two alternative combinations, we estimated the GPD for each cohort and meta-analysed the results.

We also calculated the theoretical GPD derived in the original study^18^. The theoretical value (*θ*) followed the formula,

$$\theta = \frac{{\rho f_0}}{{2 - \rho (2 - f_0)}}\left[ {1 + \frac{{M(1 - \rho )}}{{nh_{\mathrm{{eq}}}^2}}\left\{ {1 + \frac{{\rho f_0}}{{2(1 - \rho )}}} \right\}^{ - 3}} \right]^{ - 1}$$

where $\rho = rh_{\mathrm{{eq}}}^2$, *r* is a phenotypic correlation between spouses, $h_{\mathrm{eq}}^2$ is an equilibrium heritability of the phenotype, $f_0 \approx f_{\mathrm{{eq}}}/(1 - \rho )$, $f_{{\rm{eq}}} = h_{{\rm{snp}}}^2/h_{{\rm{eq}}}^2,$ $h_{{\rm{snp}}}^2$ is a SNP-based heritability, *M* is the number of causal variants and *n* is the sample size of the GWAS.

### Cross-population analysis using the UKB GWAS data

We analysed individuals of white British ancestry determined by PCA (*n* = 337,139) from UKB by adopting the tenfold LOGO approach to the six available traits (adult height, BMI, T2D, CAD, duration of light-PA and yoghurt consumption)^50^. When adult height and BMI were measured multiple times, we adopted the mean value to obtain a single value per participant and normalized the values using the rank-based inverse normal transformation method. Regarding T2D, the case was defined following the ICD-10 codes and ‘probable T2D’ and ‘possible T2D’ in a T2D inference algorithm based on Eastwood et al.^61^. We also defined individuals without any diabetes status as the T2D control based on ICD-10 and the inference algorithm. As for CAD, the case was extracted following ICD-10 codes, surgical procedure recodes, self-reported illness codes and self-reported operation codes based on Fall et al.^62^. Regarding the duration of light-PA (Data-Field 104920), we extracted the data from instance 0 (*n* = 70,692) and converted the coding to categorical values. Regarding the consumption of yoghurt, we extracted data from instance 0 within consumers of yoghurt/ice cream as binary traits (*n* = 70,692 and Data-Field 102080). From the imputed GWAS data, we excluded the variants that satisfied MAF ≤ 1% and INFO score ≤0.8, and fastGWA conducted generalized MLM approaches for nine subset samples with adjustment for age, age-squared, sex, top 20 PCs, ascertainment centre information and batch information as covariates. For the six phenotypes, we estimated the PGSs for odd and even chromosomes by PRS-CS-auto using genome-wide HapMap SNPs and the 1KG EUR LD reference panel, and the GPD was estimated in the same way as described in the Japanese study. We further meta-analysed the GPD estimate from each of the ten subgroups by the fixed effect method using metafor.

### Reporting summary

Further information on research design is available in the Nature Research Reporting Summary linked to this article.

## Supplementary information

Supplementary Information Supplementary Note, Figs. 1–6 and Tables 1, 3, 4, 6 and 7.

Reporting Summary

Supplementary Table 2 Overview of the studied traits in BBJ.

Supplementary Table 5 LD score regression results of whole-sample GWAS and variance explained by PGS in BBJ.

Supplementary Table 8 Phenotype QC in BBJ.

## Data Availability

GWAS data of the BioBank Japan Project are available at the National Bioscience Database Center (NBDC) Human Database with the research ID: hum0014 (https://humandbs.biosciencedbc.jp/hum0014-v26). GWAS data of Nagahama cohort are available at NBDC Human Database with the research ID: hum0012.v1 (https://humandbs.biosciencedbc.jp/hum0012-v1). The analysis of UKB GWAS data was conducted via the application number 47821 (https://www.ukbiobank.ac.uk/).

## Code availability

We used the publicly available software packages for data analyses. The software is described in the Methods.
