# Supplementary material for: Genetic footprints of assortative mating in the Japanese population
Source: Nat Hum Behav. 2022 Sep 22;7(1):65–73. doi: 10.1038/s41562-022-01438-z (PMC9883156; doi:10.1038/s41562-022-01438-z)
Supplement: Supplementary file 1 — Supplementary Note, Figs. 1–6 and Tables 1, 3, 4, 6 and 7. [file 41562_2022_1438_MOESM1_ESM.pdf]

nature portfolio

https://doi.org/10.1038/s41562-022-01438-z

Supplementary information

Genetic footprints of assortative mating in the Japanese population

In the format provided by the authors and unedited

Supplementary Information

Genetic footprints of assortative mating in the Japanese population

Yamamoto K et al

Corresponding to: Yukinori Okada (yokada@sg.med.osaka-u.ac.jp)

Table of Contents

Supplementary Note 2
1. Adjustment of Geographical effect in the GPD estimates in BBJ 2

Supplementary Figure 3
1. A scatter plot of principal component analysis for the BBJ participant 3
2. Scatter plots of PCA for UKB participants 4
3. A correlation between PGSs and PCs in ever vs never drinking 5
4. The associations between the number of PCs to correct population stratification and GPD estimates in the five significant traits 6
5. Forest plots of GPD estimate for the five traits with significant AM associations from other chromosome combinations 7
6. Comparison of the conventional GPD estimates and the regional GPD estimates in BBJ data 9

Supplementary Table 10
1. Characteristics of the participants in BioBank Japan Project 10
2. Overview of the studied traits in BioBank Japan Project 11
3. Characteristics of the anthropometric and biomarker traits in BioBank Japan Project . 12
4. Distributions of the dietary and behavior traits in BioBank Japan Project 14
5. LD score regression results of whole sample GWAS and variance explained by PGS in BioBank Japan Project 15
6. The meta-analyzed GPD estimates of 81 human complex traits in the Japanese population 16
7. Parameters to compute the theoretical GPDs in target traits 19
8. Phenotype quality controls in BioBank Japan Project 20

Supplementary Information 21
1. A list of all members of BioBank Japana Project 21

Supplementary Note

Adjustment of Geographical effect in the GPD estimates in BBJ

Differences in geographical distribution within Japan have been reported for various traits, for example, natto consumption is higher in eastern than in western areas of Japan. The geographical factor not captured by PCs could have an impact on partners similarities and AM. Based on the regional information defined according to registered sites grouped into the six geographical regions from the northeast to southwest of the mainland of Japan (i.e., Hokkaido [*n* = 7,645], Tohoku [*n* = 10,629], Kanto-Koshinetsu [*n* = 91,743], Chubu-Hokuriku [*n* = 9,097], Kinki [*n* = 24,044] and Kyushu [*n* = 11,754])[27], we investigated the regional difference in the 81 complex traits by ANOVA. We detected significant differences among the geographical regions in all the 81 traits. The regional difference was especially high in light PA, natto consumption, yoghurt consumption, T2D, and CAD (**Supplementary Fig. 6 (a)**).

Next, to estimate the GPD corrected by the regional difference, we adopted the leave-one-region-out (LORO) approach. Similar to the LOGO approach, we separated the individuals from the BBJ mainland into the six geographical regions. For each of the target regional groups, we conducted MLM-GWAS by fastGWA using all of the other regional samples with the adjustment for the same covariates as the LOGO approach. However, we used the top 20 PCs calculated from whole samples in BBJ mainland to control for population stratification. And then, we calculated PGS_{odd} and PGS_{even} using PRS-CS and PLINK2 and estimated the regional GPD for each trait from the correlation between PGS_{odd} and PGS_{even} regressed on top 20 PCs calculated from whole samples in BBJ mainland. Finally, we meta-analyzed the GPD estimate from each of the six regional subsets using the fixed effect method using metafor. Even after the LORO approach, the GPD estimates of both T2D and CAD remained significant, while we observed the decrease in the GPD estimates for many traits. In vegetable consumption, we detected the newly significant association. In particular, the GPD estimates of natto and yoghurt consumptions dropped to near 0 (**Supplementary Fig. 6 (b)**). From these results, the significant GPD estimates in T2D, CAD, and vegetable consumption might reflect the effect of parental AM not affected by the geographical factor.

Supplementary Figures

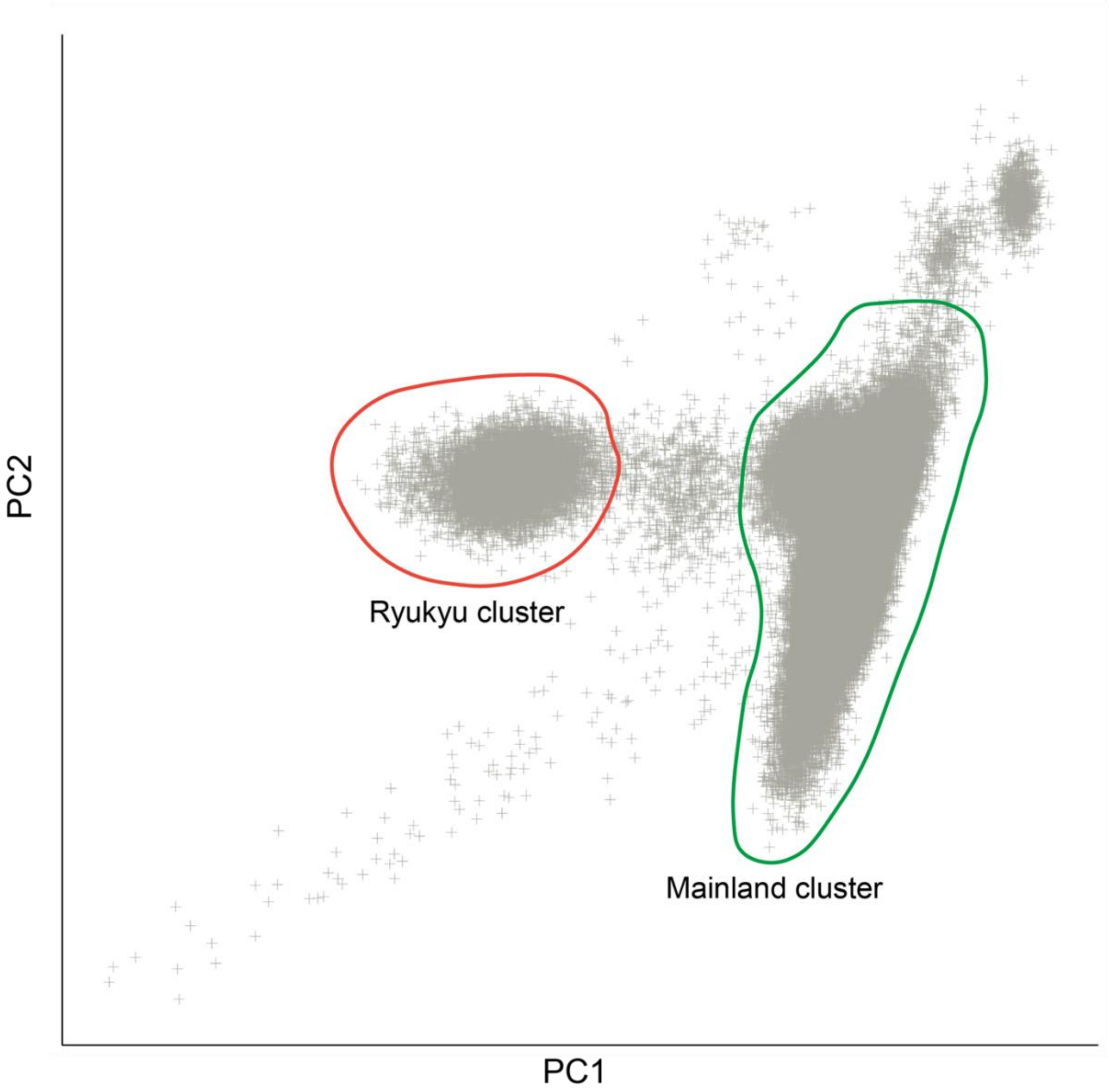

Supplementary Fig. 1. A scatter plot of principal component analysis for the BBJ participants.

We conducted PCA using PLINK2 for the BBJ participants (*n* = 166,970). We selected the individuals in the Mainland cluster (*n* = 156,151) and the Ryukyu cluster (*n* = 8,947) based on the values of PC1 and PC2.

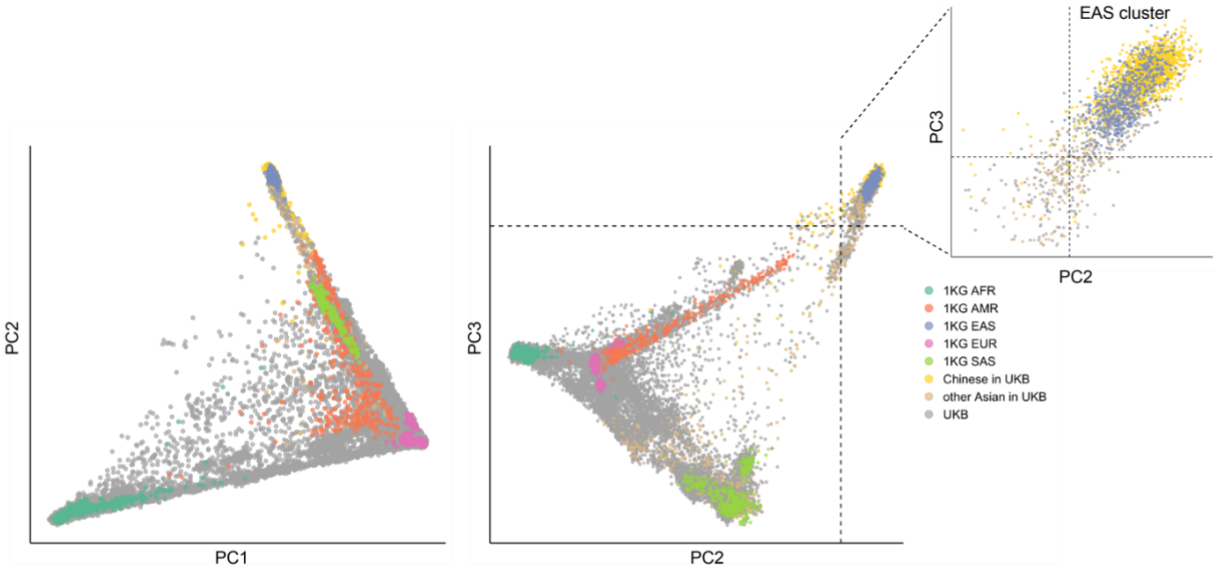

Supplementary Fig. 2. Scatter plots of PCA for UKB participants.

We conducted PCA using PLINK2 for UKB participants (n = 407,128) combining with 1KG. We selected the individuals in EAS cluster based on PCA. 1KG; 1000 Genomes Projects, AFR; African, AMR; American, EAS; East Asian, EUR; European, SAS; South East Asian, UKB; UK Biobank.

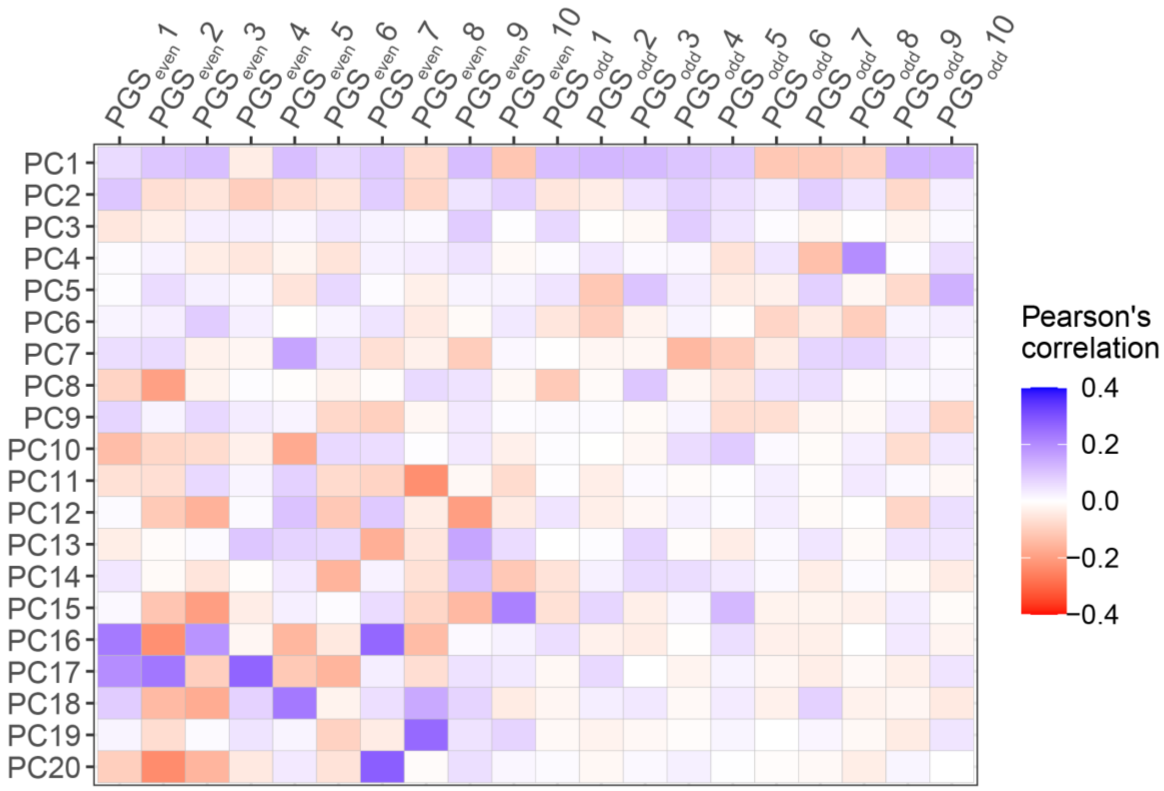

Supplementary Fig. 3. A correlation between PGSs and PCs in ever vs never drinking.

Heatmap of the correlation between PGS_{even} and 20 PCs_{even}, and PGS_{odd} and 20 PCs_{odd}. The color represents the Pearson's correlation coefficient between PGSs and each PCs across ten subsets.

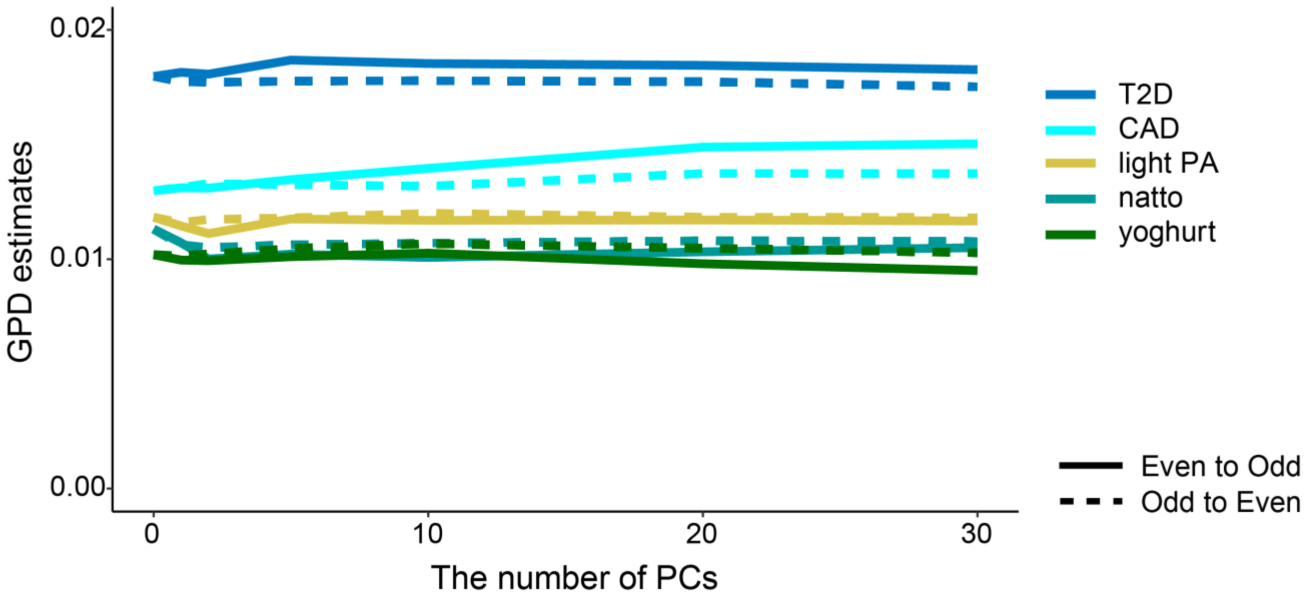

Supplementary Fig. 4. The associations between the number of PCs to correct population stratification and GPD estimates in the five significant traits.

We evaluated the impact of the number of PCs adjusted for to correct population stratification on estimate of GPD in 5 significant traits, T2D, CAD, light PA, natto consumption, yoghurt consumption. The number of PCs were changed from 0 to 30. GPD; gametic phase disequilibrium, T2D; type 2 diabetes, CAD; coronary artery disease, PA; physical activity.

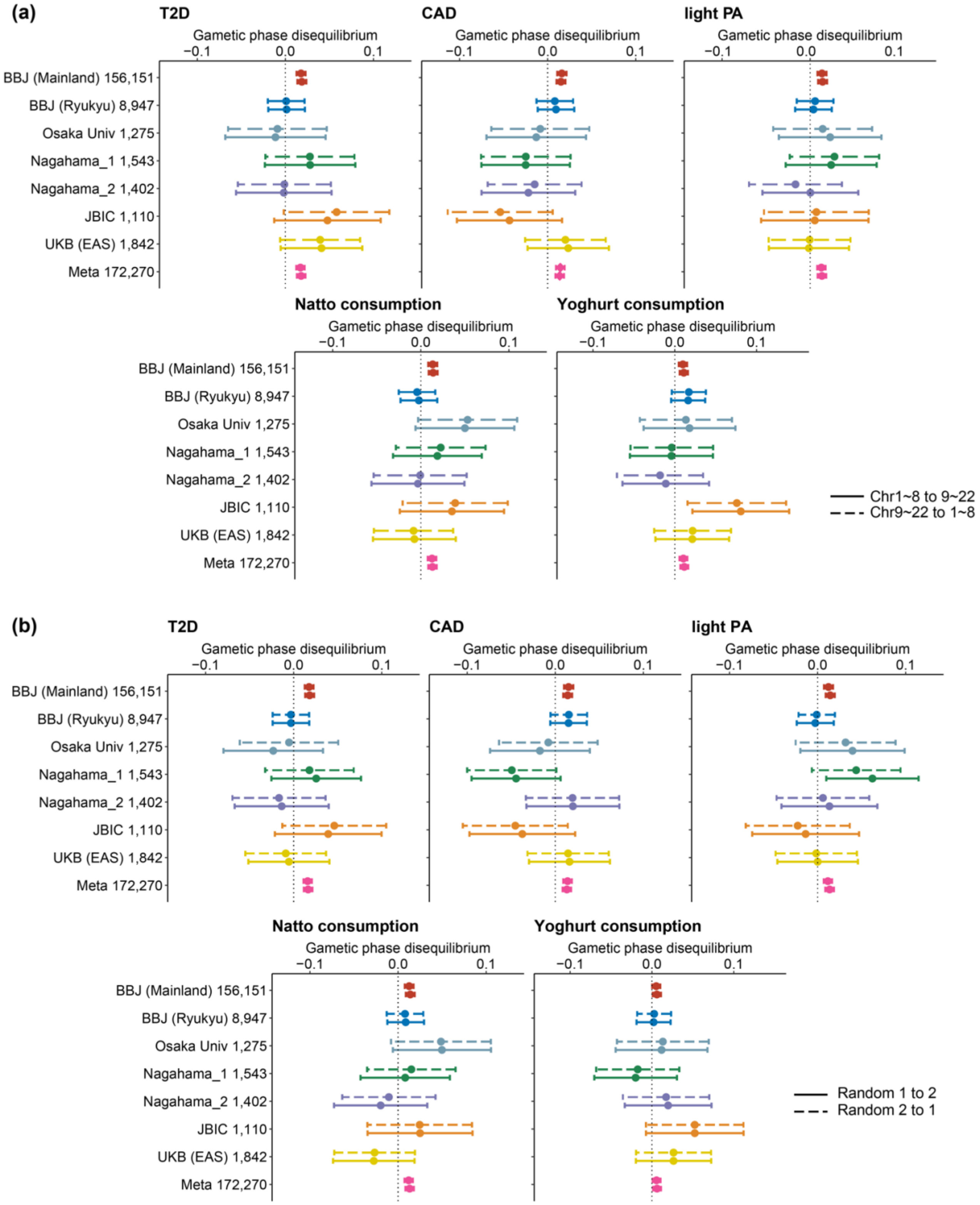

Supplementary Fig. 5. Forest plots of GPD estimate for the five traits with significant AM associations from other chromosome combinations.

We made the combinations of chromosomes so that the number of SNPs used was the same in 5 significant traits, T2D, CAD, light PA, natto consumption, yoghurt consumption**.** (a) is the forest plots of the GPD estimated from chromosome 1 to 8 and chromosome 9 to 22. θ_{T2D} =

0.018 (SE = 0.0025), θ_{CAD} = 0.014 (SE = 0.0025), $\theta_{light\ PA}$ = 0.013 (SE = 0.0025), θ_{natto} = 0.013 (SE = 0.0025), and $\theta_{yoghurt}$ = 0.010 (SE = 0.0024). (b) is the forest plot of the GPD estimated from pseudo random number chromosomes 1 (including chromosome 1, 3, 5, 6, 9, 10, 13, 14, 17 and 18) and 2 (including chromosome 2, 4, 7, 8, 11, 12, 15, 16, 19, 20, 21 and 22). θ_{T2D} = 0.016 (SE = 0.0024), θ_{CAD} = 0.014 (SE = 0.0025), $\theta_{light\ PA}$ = 0.014 (SE = 0.0025), θ_{natto} = 0.012 (SE = 0.0025), and $\theta_{yoghurt}$ = 0.0056 (SE = 0.0024). The given name was the cohort's name. The given number was the sample size of each cohort. The points indicate the point estimates, and error bars indicate 95% confidence intervals. BBJ; Biobank Japan, Osaka Univ; Osaka University healthy cohort, JBIC; Japan Biological Informatics Consortium, UKB; UK Biobank, Meta; meta-analysis, T2D; type 2 diabetes, CAD; coronary artery disease, PA; physical activity.

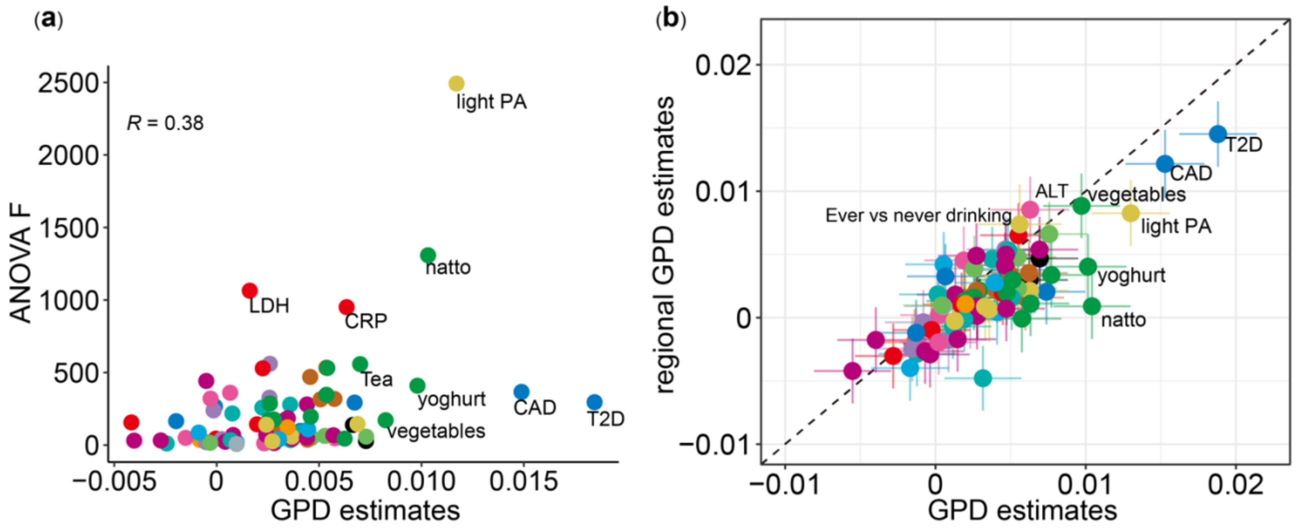

Supplementary Fig. 6. Comparison of the conventional GPD estimates and the regional GPD estimates in BBJ data

Left: co-plot of ANOVA F-values and the regional GPD estimated by the LORO approach of 81 complex traits. The x-axis indicates the conventional GPD estimates. The y-axis indicates the ANOVA F-values. Right: co-plot of the conventional GPD estimates and the regional GPD estimates of 81 complex traits. The x-axis indicates the conventional GPD estimates. The y-axis indicates the regional GPD estimates. The dotted line represents the regional GPD = the conventional GPD. T2D: type 2 diabetes, CAD; coronary artery disease, PA; physical activity, ALT; alanine aminotransferase.

Supplementary Table 1. Characteristics of the participants in BioBank Japan project

BioBank Japan	Male		Female	
	N	%	N	%
Sex	84,215	54.2	71,091	45.8
Age group at entry (years)				
<20	89	52.4	81	47.6
20-29	1,619	43.3	2,123	56.7
30-39	3,792	44.8	4,676	55.2
40-49	6,392	47.2	7,141	52.8
50-59	15,585	55.0	12,744	45.0
60-69	26,013	57.7	19,034	42.3
70-79	24,328	56.7	18,590	43.3
≥80	6,397	48.8	6,702	51.2

Mean age at recruitment	63.0

Supplementary Table 2. Overview of the studied traits in BioBank Japan project

(Results are indicated in a separate Microsoft Excel file)

Supplementary Table 3. Characteristics of the quantitative traits in BioBank Japan project

Trait	Mean	Median	Standard deviation	Unit
Height	159.9	160.0	9.0	cm
BMI	23.1	22.9	3.5	kg/m^2
TC	208.1	205.0	45.3	mg/dL
HDL-C	54.8	53.0	15.5	mg/dL
LDL-C	130.6	126.6	41.1	mg/dL
TG	132.6	115.0	71.8	mg/dL
BS	106.5	101.0	21.2	mg/dL
HbA1c	5.5	5.4	0.6	%
TP	7.1	7.1	0.6	g/dL
Alb	4.2	4.3	0.4	g/dL
BUN	15.5	15.0	4.9	mg/dL
sCr	0.9	0.8	1.0	mg/dL
eGFR	73.6	75.2	15.8	ml/min/1.73m^2
UA	5.4	5.3	1.5	mg/dL
Na	141.2	141.0	2.6	mEq/L
K	4.3	4.2	0.4	mEq/L
Cl	104.5	105.0	2.8	mEq/L
Ca	9.2	9.2	0.5	mg/dL
P	3.4	3.4	0.6	mg/dL
TBil	0.6	0.6	0.3	mg/dL
AST	22.9	21.0	7.8	IU/L
ALT	22.1	19.0	11.9	IU/L
ALP	234.4	223.0	83.1	IU/L
GGT	34.3	26.0	24.6	IU/L
PT	1.0	1.0	0.1	sec
APTT	30.1	29.1	4.7	sec
Fbg	322.9	305.9	104.1	mg/dL
CK	101.0	87.0	56.7	IU/L
LDH	224.3	197.0	90.5	IU/L
CRP	0.3	0.1	0.3	mg/dL
WBC	6127.6	5860.0	1882.1	/μl
Neutro	3646.8	3403.8	1429.5	/μl
Eosino	171.1	133.2	136.5	/μl
Baso	34.3	29.5	25.1	/μl
Mono	373.4	346.8	166.0	/μl
Lym	1786.0	1711.9	718.8	/μl
RBC	435.3	437.0	54.5	10^4/μl

Hb	13.4	13.5	1.7	g/dL
Ht	40.3	40.5	4.8	%
MCV	92.9	92.9	5.2	fL
MCH	31.0	31.1	2.0	pg
MCHC	33.4	33.4	1.1	%
Plt	22.6	21.9	6.8	10^4/μl
SBP	134.7	135.0	19.8	mmHg
DBP	79.4	80.0	12.2	mmHg
MAP	97.8	97.3	13.6	mmHg
PP	55.3	55.0	14.0	mmHg
IVS	9.9	10.0	2.0	mm
PW	9.8	10.0	1.7	mm
LVDd	48.9	48.0	7.3	mm
LVDs	32.5	31.0	8.1	mm
LVM	174.5	167.4	54.0	g
LVMI	106.3	101.5	30.8	g/m^2
RWT	0.4	0.4	0.1	[ratio]
FS	0.3	0.4	0.1	%
EF	61.2	64.0	13.3	%
E/A	0.9	0.8	0.3	[ratio]

Supplementary Table 4. Distributions of the dietary and behavior traits in BioBank Japan project

Dietary habit

Trait	Rarely (0)	1-2 day/week (1.5)	3-4 day/week (3.5)	Everyday (7)	Total n
Cheese	79,058	39,636	11,784	10,717	141,195
Coffee	44,586	13,723	9,351	73,453	141,113
Fish	3,586	26,238	68,871	42,838	141,533
Meat	17,061	57,187	49,759	17,340	141,347
Milk	47,998	20,037	15,512	57,890	141,437
Natto	41,562	38,059	30,050	31,496	141,167
Small fish	30,671	47,017	32,298	30,834	140,820
Green tea	15,306	6,301	6,259	113,272	141,138
Tofu	8,559	42,131	48,150	42,563	141,403
Vegetables	3,034	9,553	20,224	108,663	141,474
Yoghurt	51,966	30,514	18,110	40,808	141,398

Behavior trait

Trait	Rarely (0)	1-2 times a week less than 15/30 min (15/30)	1-2 times a week for 15/30 min or more (22.5/45)	3-4 times a week for less than 15/30 min (35/70)	3-4 times a week for 15/30 min or more (52.5/105)	Everyday for less than 15/30 min (70/140)	Everyday for 15/30 min or more (105/210)	Total n
Gym	105,633	3,350	4,731	2,263	2,422	12,125	9,109	139,633
Sports	115,144	1,068	13,145	287	4,478	598	4,269	138,989
Walking	73,412	2,765	8,722	1,805	8,056	7,695	38,886	141,341
light PA	24,737	3,460	13,440	2,517	15,172	7,247	74,534	141,107

Supplementary Table 5. LD score regression results of whole sample GWAS and variance explained by PGS in BioBank Japan project

(Results are indicated in a separate Microsoft Excel file)

Supplementary Table 6. The meta-analyzed GPD estimates of 81 human complex traits in the Japanese population

Abbreviation	Category	GPD estimate	SE	*P*-value	P_{Het}
**T2D**	**Disease**	0.0185	0.0025	5.2×10^{-14}	0.01
**CAD**	**Disease**	0.0149	0.0025	2.2×10^{-9}	0.14
**light PA**	**Behavior**	0.0117	0.0025	2.0×10^{-6}	0.18
**Natto**	**Diet**	0.0103	0.0024	2.4×10^{-5}	0.64
**Yoghurt**	**Diet**	0.0098	0.0024	5.6×10^{-5}	0.67
**Vegetables**	**Diet**	0.0083	0.0024	6.9×10^{-4}	0.34
**TC**	**Metabolic**	0.0073	0.0024	2.7×10^{-3}	0.36
**Height**	**Anthropometric**	0.0073	0.0024	2.7×10^{-3}	0.79
**Green tea**	**Diet**	0.0070	0.0024	4.1×10^{-3}	0.54
**Gym**	**Behavior**	0.0069	0.0024	4.7×10^{-3}	0.65
**Dyslipidemia**	**Disease**	0.0067	0.0024	5.9×10^{-3}	0.25
**BMI**	**Anthropometric**	0.0067	0.0024	6.2×10^{-3}	0.9
**CRP**	**Other biochemical**	0.0063	0.0024	0.01	0.94
**Milk**	**Diet**	0.0062	0.0024	0.01	0.15
**ALT**	**Liver-related**	0.0058	0.0025	0.02	0.03
**PP**	**Blood pressure**	0.0057	0.0024	0.02	0.37
**MCV**	**Hematological**	0.0057	0.0024	0.02	0.32
**Alcohol**	**Behavior**	0.0055	0.0026	0.04	0.67
**Tofu**	**Diet**	0.0054	0.0025	0.03	0.50
**Fish**	**Diet**	0.0054	0.0025	0.03	0.93
**SBP**	**Blood pressure**	0.0054	0.0025	0.03	0.11
**HbA1c**	**Metabolic**	0.0053	0.0024	0.03	0.33
**DBP**	**Blood pressure**	0.0051	0.0025	0.04	0.61
**HDLC**	**Metabolic**	0.0046	0.0025	0.06	0.50
**Small fish**	**Diet**	0.0046	0.0024	0.06	0.56
**MAP**	**Blood pressure**	0.0046	0.0025	0.06	0.45
**eGFR**	**Kidney-related**	0.0045	0.0024	0.07	0.48
**LVDs**	**Blood pressure**	0.0044	0.0024	0.07	0.76
**LVMI**	**Echocardiographic**	0.0044	0.0024	0.07	0.01
**Mono**	**Hematological**	0.0044	0.0024	0.07	0.51

**WBC**	**Hematological**	0.0044	0.0024	0.07	0.33
**LVM**	**Echocardiographic**	0.0041	0.0024	0.09	0.15
**Arrhythmia**	**Disease**	0.0041	0.0024	0.09	0.006
**Lym**	**Hematological**	0.0037	0.0024	0.13	0.15
**Smoking**	**Behavior**	0.0037	0.0025	0.14	0.96
**EF**	**Echocardiographic**	0.0036	0.0024	0.14	0.33
**IVS**	**Echocardiographic**	0.0036	0.0024	0.14	0.99
**MCH**	**Hematological**	0.0035	0.0024	0.16	0.55
**TP**	**Protein**	0.0035	0.0025	0.16	0.07
**BS**	**Metabolic**	0.0034	0.0024	0.16	0.46
**PLT**	**Hematological**	0.0034	0.0025	0.18	0.15
**UA**	**Kidney-related**	0.0030	0.0025	0.21	0.37
**LDLC**	**Metabolic**	0.0029	0.0024	0.23	0.63
**LVDd**	**Blood pressure**	0.0029	0.0024	0.23	0.81
**Neutro**	**Hematological**	0.0028	0.0024	0.25	0.85
**Meat**	**Diet**	0.0028	0.0024	0.25	0.27
**Sports**	**Behavior**	0.0027	0.0024	0.26	0.57
**Cheese**	**Diet**	0.0026	0.0024	0.28	0.68
**Cl**	**Electrolyte**	0.0026	0.0024	0.29	0.05
**Na**	**Electrolyte**	0.0026	0.0024	0.29	0.57
**Coffee**	**Diet**	0.0026	0.0025	0.31	0.19
**Walking**	**Behavior**	0.0024	0.0024	0.32	0.76
**RBC**	**Hematological**	0.0024	0.0024	0.32	0.17
**AST**	**Liver-related**	0.0023	0.0025	0.35	0.06
**PTINR**	**Other biochemical**	0.0023	0.0024	0.36	0.06
**RWT**	**Echocardiographic**	0.0022	0.0024	0.36	0.10
**Fbg**	**Other biochemical**	0.0020	0.0024	0.42	0.40
**LDH**	**Other biochemical**	0.0016	0.0024	0.51	0.57
**BUN**	**Kidney-related**	0.0010	0.0024	0.69	0.79
**Dummy**	**Control**	0.0010	0.0024	0.69	0.78
**Baso**	**Hematological**	0.0008	0.0024	0.74	0.58
**PW**	**Echocardiographic**	0.0008	0.0024	0.75	0.74
**FS**	**Echocardiographic**	0.0007	0.0024	0.78	0.36
**ALP**	**Liver-related**	0.0007	0.0024	0.79	0.32

**Eosino**	**Hematological**	0.0004	0.0025	0.87	0.78
**Ca**	**Electrolyte**	0.0003	0.0024	0.92	0.04
**CK**	**Other biochemical**	0.0000	0.0024	0.99	0.74
**Cataract**	**Disease**	-0.0001	0.0024	0.97	0.16
**K**	**Electrolyte**	-0.0002	0.0024	0.95	0.70
**TBil**	**Liver-related**	-0.0003	0.0024	0.90	0.57
**TG**	**Metabolic**	-0.0003	0.0024	0.90	0.05
**MCHC**	**Hematological**	-0.0005	0.0024	0.83	0.82
**P**	**Electrolyte**	-0.0005	0.0024	0.83	0.26
**Alb**	**Protein**	-0.0008	0.0025	0.74	0.68
**sCr**	**Kidney-related**	-0.0009	0.0025	0.72	0.56
**GGT**	**Liver-related**	-0.0015	0.0025	0.54	0.88
**IS**	**Disease**	-0.0020	0.0024	0.42	0.86
**E/A**	**Echocardiographic**	-0.0024	0.0024	0.32	0.12
**Ht**	**Hematological**	-0.0027	0.0024	0.26	0.34
**Hb**	**Hematological**	-0.0040	0.0024	0.10	0.37
**APTT**	**Other biochemical**	-0.0042	0.0024	0.09	0.16

* *P*-values were determined by two-sided Wald test.
* The red line indicates the study-wide significant threshold at *P* = 6.1 × 10^-4 (= 0.05/81) by applying the Bonferroni correction for multiple comparison.
* P_{Het} is *P* value of heterogeneity among cohorts calculated by cochrane's Q test (two-sided)

Supplementary Table 7. Parameters to compute the theoretical GPDs in target traits

**Trait**	**mate correlation (*r*)†**	**mate correlation (odds ratio)†**	**heritability (*heq*)†**	**SNP heritability by GREML-LDMS (*hsnp*, in 50,000 samples)**	**sample size (*N*)**	**Causal variant (*M*)**	**thetaSnps**
Dummy	0.0001‡	-	0.5	0.5	150,000	10,000~1,000,000	0.00002~0.0002
Height	0.175	-	0.68	0.4	150,000	10,000~1,000,000	0.0048~0.038
BMI	0.136	-	0.8	0.2	150,000	10,000~1,000,000	0.0021~0.016
T2D	0.1§	1.34	0.5	0.2	150,000	10,000~1,000,000	0.0098~0.0008
CAD	0.1§	1.35	0.5	0.1	150,000	10,000~1,000,000	0.0055~0.0005

†: We extracted mate correlation and equilibrium heritability from published journals for target traits other than dummy phenotype.

‡: We set this value since the dummy phenotype was a random variable and has no mate correlation.

§: We converted odds ratio in a binary trait to Pearson's *r* via cohen's d.

Supplementary Table 8. Phenotype quality controls in BioBank Japan project

(Results are indicated in a separate Microsoft Excel file)

A list of all members of BioBank Japan Project

Koichi Matsuda.
Laboratory of Genome Technology, Human Genome Center, Institute of Medical Science, The University of Tokyo, Tokyo, Japan.
Laboratory of Clinical Genome Sequencing, Graduate School of Frontier Sciences, The University of Tokyo, Tokyo, Japan.

Yuji Yamanashi.
Division of Genetics, The Institute of Medical Science, The University of Tokyo, Tokyo, Japan.

Yoichi Furukawa.
Division of Clinical Genome Research, Institute of Medical Science, The University of Tokyo, Tokyo, Japan.

Takayuki Morisaki.
Division of Molecular Pathology, IMSUT Hospital Department of Internal Medicine, Institute of Medical Science, The University of Tokyo, Tokyo, Japan.

Yoshinori Murakami.
Department of Cancer Biology, Institute of Medical Science, The University of Tokyo, Tokyo, Japan.

Yoichiro Kamatani.
Laboratory of Complex Trait Genomics, Graduate School of Frontier Sciences, The University of Tokyo, Tokyo, Japan.

Kaori Muto.
Department of Public Policy, Institute of Medical Science, The University of Tokyo, Tokyo, Japan.

Akiko Nagai.
Department of Public Policy, Institute of Medical Science, The University of Tokyo, Tokyo, Japan.

Wataru Obara.
Department of Urology, Iwate Medical University, Iwate, Japan.

Ken Yamaji.
Department of Internal Medicine and Rheumatology, Juntendo University Graduate School of Medicine, Tokyo, Japan.

Kazuhisa Takahashi.
Department of Respiratory Medicine, Juntendo University Graduate School of Medicine, Tokyo, Japan.

Satoshi Asai.
Division of Pharmacology, Department of Biomedical Science, Nihon University School of Medicine, Tokyo, Japan.

Division of Genomic Epidemiology and Clinical Trials, Clinical Trials Research Center, Nihon University. School of Medicine, Tokyo, Japan.

Yasuo Takahashi.
Division of Genomic Epidemiology and Clinical Trials, Clinical Trials Research Center, Nihon University School of Medicine, Tokyo, Japan.

Takao Suzuki.
Tokushukai Group, Tokyo, Japan.

Nobuaki Sinozaki.
Tokushukai Group, Tokyo, Japan.

Hiroki Yamaguchi.
Departmentof Hematology, Nippon Medical School, Tokyo, Japan.

Shiro Minami.
Department of Bioregulation, Nippon Medical School, Kawasaki, Japan.

Shigeo Murayama.
Tokyo Metropolitan Geriatric Hospital and Institute of Gerontology, Tokyo, Japan.

Kozo Yoshimori.
Fukujuji Hospital, Japan Anti-Tuberculosis Association, Tokyo, Japan.

Satoshi Nagayama.
The Cancer Institute Hospital of the Japanese Foundation for Cancer Research, Tokyo, Japan.

Daisuke Obata.
Center for Clinical Research and Advanced Medicine, Shiga University of Medical Science, Shiga, Japan.

Masahiko Higashiyama.
Department of General Thoracic Surgery, Osaka International Cancer Institute, Osaka, Japan.

Akihide Masumoto.
IIZUKA HOSPITAL, Fukuoka, Japan.

Yukihiro Koretsune.
National Hospital Organization Osaka National Hospital, Osaka, Japan.
