# Supplementary material for: Genetic footprints of assortative mating in the Japanese population
Source: Nat Hum Behav. 2022 Sep 22;7(1):65–73. doi: 10.1038/s41562-022-01438-z (PMC9883156; doi:10.1038/s41562-022-01438-z)
Supplement: Supplementary file 2 — Reporting Summary [file 41562_2022_1438_MOESM2_ESM.pdf]

nature portfolio

Corresponding author(s): Yukinori Okada

Last updated by author(s): July, 12, 2022

Reporting Summary

Nature Portfolio wishes to improve the reproducibility of the work that we publish. This form provides structure for consistency and transparency in reporting. For further information on Nature Portfolio policies, see our Editorial Policies and the Editorial Policy Checklist.

Statistics

For all statistical analyses, confirm that the following items are present in the figure legend, table legend, main text, or Methods section.

n/a	Confirmed	
☐	☒	The exact sample size (n) for each experimental group/condition, given as a discrete number and unit of measurement
☐	☒	A statement on whether measurements were taken from distinct samples or whether the same sample was measured repeatedly
☐	☒	The statistical test(s) used AND whether they are one- or two-sided<br>*Only common tests should be described solely by name; describe more complex techniques in the Methods section.*
☐	☒	A description of all covariates tested
☐	☒	A description of any assumptions or corrections, such as tests of normality and adjustment for multiple comparisons
☐	☒	A full description of the statistical parameters including central tendency (e.g. means) or other basic estimates (e.g. regression coefficient) AND variation (e.g. standard deviation) or associated estimates of uncertainty (e.g. confidence intervals)
☐	☒	For null hypothesis testing, the test statistic (e.g. F, t, r) with confidence intervals, effect sizes, degrees of freedom and P value noted<br>*Give P values as exact values whenever suitable.*
☐	☒	For Bayesian analysis, information on the choice of priors and Markov chain Monte Carlo settings
☒	☐	For hierarchical and complex designs, identification of the appropriate level for tests and full reporting of outcomes
☐	☒	Estimates of effect sizes (e.g. Cohen's d, Pearson's r), indicating how they were calculated

Our web collection on statistics for biologists contains articles on many of the points above.

Software and code

Policy information about availability of computer code

Data collection	We did not use any software for data collection.
Data analysis	We used publicly available software for the data analysis (PLINK (v1.90b4.4), PLINK2 (v2.00a2.3 and v2.00a3), R 3.4.0, Eagle (v2), Minimac3 (2.0.1), Shapeit (v2), GCTA (version 1.93.2beta and 1.93.2beta2), IMPUTE4, LDSC (v1.0.0), PRS-CS (original version), metafor (v1.9-9)). The software is described in the Methods section of the manuscript.

For manuscripts utilizing custom algorithms or software that are central to the research but not yet described in published literature, software must be made available to editors and reviewers. We strongly encourage code deposition in a community repository (e.g. GitHub). See the Nature Portfolio guidelines for submitting code & software for further information.

Data

Policy information about availability of data

All manuscripts must include a data availability statement. This statement should provide the following information, where applicable:

- Accession codes, unique identifiers, or web links for publicly available datasets
- A description of any restrictions on data availability
- For clinical datasets or third party data, please ensure that the statement adheres to our policy

GWAS data of the BioBank Japan Project (BBJ) are available at the NBDC Human Database with the research ID: hum0014 (https://humandbs.biosciencedbc.jp/hum0014-v26). GWAS data of Nagahama cohort are available at NBDC Human Database with the research ID: hum0012.v1 (https://humandbs.biosciencedbc.jp/hum0012-v1). The analysis of UK Biobank (UKB) GWAS data was conducted via the application number 47821 (https://www.ukbiobank.ac.uk/).

Field-specific reporting

Please select the one below that is the best fit for your research. If you are not sure, read the appropriate sections before making your selection.

☒ Life sciences ☐ Behavioural & social sciences ☐ Ecological, evolutionary & environmental sciences

For a reference copy of the document with all sections, see nature.com/documents/nr-reporting-summary-flat.pdf

Life sciences study design

All studies must disclose on these points even when the disclosure is negative.

Sample size	Clinical information and genotype data were obtained from BBJ, which is a biobank that collaboratively collected DNA and serum samples from 12 medical institutions in Japan and recruited approximately 200,000 participants. UKB is a population-based prospective cohort that recruited approximately 500,000 people. Sample size calculation was not carried out, but we used maximum samples in BBJ, whose phenotype data were available.
Data exclusions	In BBJ, we excluded individuals with the age under 18, low call rate in genotyping (< 98%), closely related (PI_HAT < 0.125), and ancestry other than Japanese (based on PCA plot) for quality control as described in Sakaue et al Nat Med 2020, Akiyama et al. Nat Commun 2019, and Kanai et al. Nat Genet 2018. We extracted the individuals into the mainland cluster by visual inspection based on the PCA plot of BBJ (n = 156,151).
Replication	We replicated our findings of assortative mating by conducting the same analysis with the same pipeline in six Japanese and East Asian cohorts (n = 16,119) and UK Biobank cohort (n = 337,139).
Randomization	In leave-one-group-out method, we randomly split the whole participants into 10 groups.
Blinding	We did not apply blinding of the samples because our study was an observational study and no intervention was conducted in our study.

Reporting for specific materials, systems and methods

We require information from authors about some types of materials, experimental systems and methods used in many studies. Here, indicate whether each material, system or method listed is relevant to your study. If you are not sure if a list item applies to your research, read the appropriate section before selecting a response.

Materials & experimental systems

n/a	Involved in the study	
☒	☐	Antibodies
☒	☐	Eukaryotic cell lines
☒	☐	Palaeontology and archaeology
☒	☐	Animals and other organisms
☐	☒	Human research participants
☒	☐	Clinical data
☒	☐	Dual use research of concern

Methods

n/a	Involved in the study	
☒	☐	ChIP-seq
☒	☐	Flow cytometry
☒	☐	MRI-based neuroimaging

Human research participants

Policy information about studies involving human research participants

Population characteristics	BBJ is a hospital-based cohort, and participants have the diagnosis of at least one of 47 common diseases. The detailed information of participants such as age and sex distributions is summarized in Supplementary Table 1. UKB is a population-based cohort, enrolling healthy volunteers aged between 40 and 69 years. Mean age of participants was 56.9 years old, and the ratio of female was 53.7%.
Recruitment	BBJ collaboratively collected DNA and serum samples from 12 medical institutions in Japan and recruited approximately 200,000 participants with the diagnosis of at least one of 47 diseases from 2003 to 2008. UKB is a population-based cohort study that recruited approximately 500,000 individuals from 2006 to 2010 from across the United Kingdom.
Ethics oversight	All the participants provided written informed consent approved from ethics committees of RIKEN Center for Integrative Medical Sciences, and the Institute of Medical Sciences, the University of Tokyo. This study was approved by the ethical committee of Osaka University Graduate School of Medicine.

Note that full information on the approval of the study protocol must also be provided in the manuscript.
